# Optical Properties of Al-Doped ZnO Films in the Infrared Region and Their Absorption Applications

**DOI:** 10.1186/s11671-018-2563-9

**Published:** 2018-05-12

**Authors:** Hua Zheng, Rong-Jun Zhang, Da-Hai Li, Xin Chen, Song-You Wang, Yu-Xiang Zheng, Meng-Jiao Li, Zhi-Gao Hu, Ning Dai, Liang-Yao Chen

**Affiliations:** 1 Key Laboratory of Micro and Nano Photonic Structures, Ministry of Education, Department of Optical Science and Engineering, Fudan University, Shanghai, 200433 China; 2 National Laboratory for Infrared Physics, Shanghai Institute of Technical Physics, Chinese Academy of Sciences, Shanghai, 200083 China; 3 Key Laboratory of Polar Materials and Devices, Ministry of Education, Department of Electronic Engineering, East China Normal University, Shanghai, 200241 China

**Keywords:** Aluminum-doped zinc oxide, Infrared, Spectroscopic ellipsometry, Absorber

## Abstract

The optical properties of aluminum-doped zinc oxide (AZO) thin films were calculated rapidly and accurately by point-by-point analysis from spectroscopic ellipsometry (SE) data. It was demonstrated that there were two different physical mechanisms, i.e., the interfacial effect and crystallinity, for the thickness-dependent permittivity in the visible and infrared regions. In addition, there was a blue shift for the effective plasma frequency of AZO when the thickness increased, and the effective plasma frequency did not exist for AZO ultrathin films (< 25 nm) in the infrared region, which demonstrated that AZO ultrathin films could not be used as a negative index metamaterial. Based on detailed permittivity research, we designed a near-perfect absorber at 2–5 μm by etching AZO-ZnO alternative layers. The alternative layers matched the phase of reflected light, and the void cylinder arrays extended the high absorption range. Moreover, the AZO absorber demonstrated feasibility and applicability on different substrates.

## Background

Plasmonics [1] and metamaterials [2] have attracted much attention in recent decades. Many unconventional functionalities, such as negative refractive index materials [3], sub-diffraction imaging [4], and invisibility cloaks [5], were presented, which conventionally used noble metals as the primary plasmonic building blocks of optical metamaterials [6]. Compared with noble metals, heavily doped semiconductors, such as aluminum-doped zinc oxide (AZO) [7] and titanium nitride (TiN) [8], have recently played a more important part in plasmonics and metamaterial applications because of their tunable free carrier concentrations. The doping density [8], growth atmosphere, and the growth or annealing temperature [9] were the usual methods to adjust the properties of heavily doped semiconductors. As a heavily doped semiconductor with broad band gap, AZO is a tunable, low-loss plasmonic material capable of supporting high dopant concentrations, and it plays an important role in plasmonic structures [10]. For example, a material system such as zinc oxide (ZnO) and AZO has an evident advantage as a result of the epitaxial and superlattice design of the device structure, which can reduce the losses at the layer interfaces and thus further boost the device performance [11–16]. Although many papers [17, 18] have focused on the properties of AZO in the visible or near-infrared region, only a few have concentrated on the infrared properties of AZO, which influence the realistic applications. Recently, Uprety et al. [19] discussed the optical properties of bulk AZO by means of recombination model simulation of spectroscopic ellipsometry (SE). The simulation was general but not rapid or convenient. In this paper, we calculated the permittivity of AZO thin films from 210 to 5000 nm by means of point-by-point analysis [20], a calculation dependent on primary SE simulation, which is a rapid and accurate method. In addition, we discussed the reasons for the thickness-dependent properties of AZO thin films in the visible and infrared bands with two different mechanisms, respectively. The thickness dependence of the band gap and effective plasma frequency of AZO were also demonstrated. We found the effective plasma frequency does not exist with low thickness (< 25 nm) in the infrared region. Furthermore, we used finite difference time domain (FDTD) solutions to design two void cylinder arrays based on AZO alternative layers, which demonstrated near perfect absorption in infrared broadband.

## Methods

Since existing atomic layer deposition (ALD) shows ultrahigh conformity and compatibility to semiconductor processing [21], it is a powerful tool for plasmonic material deposition with precisely controlled thickness. AZO thin films were deposited on p-type Si (100) by alternating diethylzinc (Zn(CH_2_CH_3_)_2_, DEZ; Al(CH_3_)_3_, TMA) and deionized water (H_2_O) in an ALD reactor (Picosun) at 190 °C. A typical ALD cycle for AZO consisted of 14 single cycles ZnO and 1 single cycle Al-O, while the single cycle of ZnO or Al-O consisted of 0.1 s DEZ or TMA pulse, 5 s N_2_ purge, 0.1 s H_2_O pulse, and 5 s N_2_ purge according to our previous reports [22–24]. The mechanism of ZnO ALD is the chemical vapor deposition reaction.

1 $$ \mathrm{Zn}{\left({\mathrm{CH}}_2{\mathrm{CH}}_3\right)}_2+{\mathrm{H}}_2\mathrm{O}\to \mathrm{Zn}\mathrm{O}+{2\mathrm{C}}_2{\mathrm{H}}_6 $$

There are two reactions in an ALD cycle.

2 $$ {\mathrm{ZnOH}}^{\ast }+\mathrm{Zn}{\left({\mathrm{C}\mathrm{H}}_2{\mathrm{C}\mathrm{H}}_3\right)}_2\to \mathrm{ZnOZn}{\left({\mathrm{C}\mathrm{H}}_2{\mathrm{C}\mathrm{H}}_3\right)}^{\ast }+{\mathrm{C}}_2{\mathrm{H}}_6 $$

3 $$ \mathrm{Zn}{\left({\mathrm{C}\mathrm{H}}_2{\mathrm{C}\mathrm{H}}_3\right)}^{\ast }+{\mathrm{H}}_2\mathrm{O}\to {\mathrm{ZnOH}}^{\ast }+{\mathrm{C}}_2{\mathrm{H}}_6 $$

And the Al doping is similar, where the cycle of Zn:Al is 14:1.

4 $$ {\mathrm{AlOH}}^{\ast }+\mathrm{Al}{\left({\mathrm{CH}}_3\right)}_3\to \mathrm{AlOAl}{{\left({\mathrm{CH}}_3\right)}_2}^{\ast }+{\mathrm{CH}}_4 $$

5 $$ \mathrm{AlOAl}{{\left({\mathrm{CH}}_3\right)}_2}^{\ast }+{2\mathrm{H}}_2\mathrm{O}\to {\mathrm{AlOAlOH}}^{\ast }+{2\mathrm{CH}}_4 $$

where * indicates a surface species.

Here, the thickness of AZO thin films was varied by controlling ALD cycles. There were three types of samples: 150, 300, and 450 cycles (here we used the fundamental single cycle as the unit of measurement). The thicknesses and optical properties of ZnO ultrathin films were obtained by a spectroscopic ellipsometer (J.A. Woollam, USA). The incident angle was fixed at 65° and the wavelength ranged from 210 to 1000 nm, 1000 to 2000 nm, and 2000 to 5000 nm. The reflection and transmission of AZO films were obtained by Fourier transform infrared spectroscopy (FTIR) measurements. X-ray diffraction (XRD) patterns suggested the optical properties changed with the thickness of the AZO films.

## Results and Discussions

### Optical Properties of AZO Films in Visible and Infrared Broadband

Due to low interfacial roughness by ALD, the monolayer model was used to describe the AZO thin films [10]. Then, the refractive index *n*, extinction coefficient *k* and thickness *d* of the resulting AZO thin films were obtained by the SE measurement. During the SE measurement [25, 26], the elliptically polarized light, carrying the material’s information after reflected by AZO films, was detected by the ellipsometer. The wavelength of the incident light was within the range of 210–5000 nm. There are two measurement parameters acquired from the polarized light, i.e., amplitude ratio (*Ψ*) and phase shift (*Δ*), which were defined by the ellipsometric ratio *ρ* as [27]:

6 $$ \rho =\frac{r_p}{r_s}=\tan \varPsi {e}^{j\Delta  } $$

Here *r*_*p*_ and *r*_*s*_ are the complex reflection coefficients of polarized light parallel and perpendicular to the incidence plane, respectively. For SE fittings, the root mean square error (RMSE) is minimized to obtain an accuracy fitting:

7 $$ \mathrm{RMSE}=\sqrt{\frac{1}{2x-y-1}\sum \limits_{i=1}^x\left[{\left({\varPsi}_i^{cal}-{\varPsi}_i^{exp}\right)}^2+{\left({\Delta }_i^{cal}-{\Delta }_i^{exp}\right)}^2\right]} $$

**Fig. 1:**
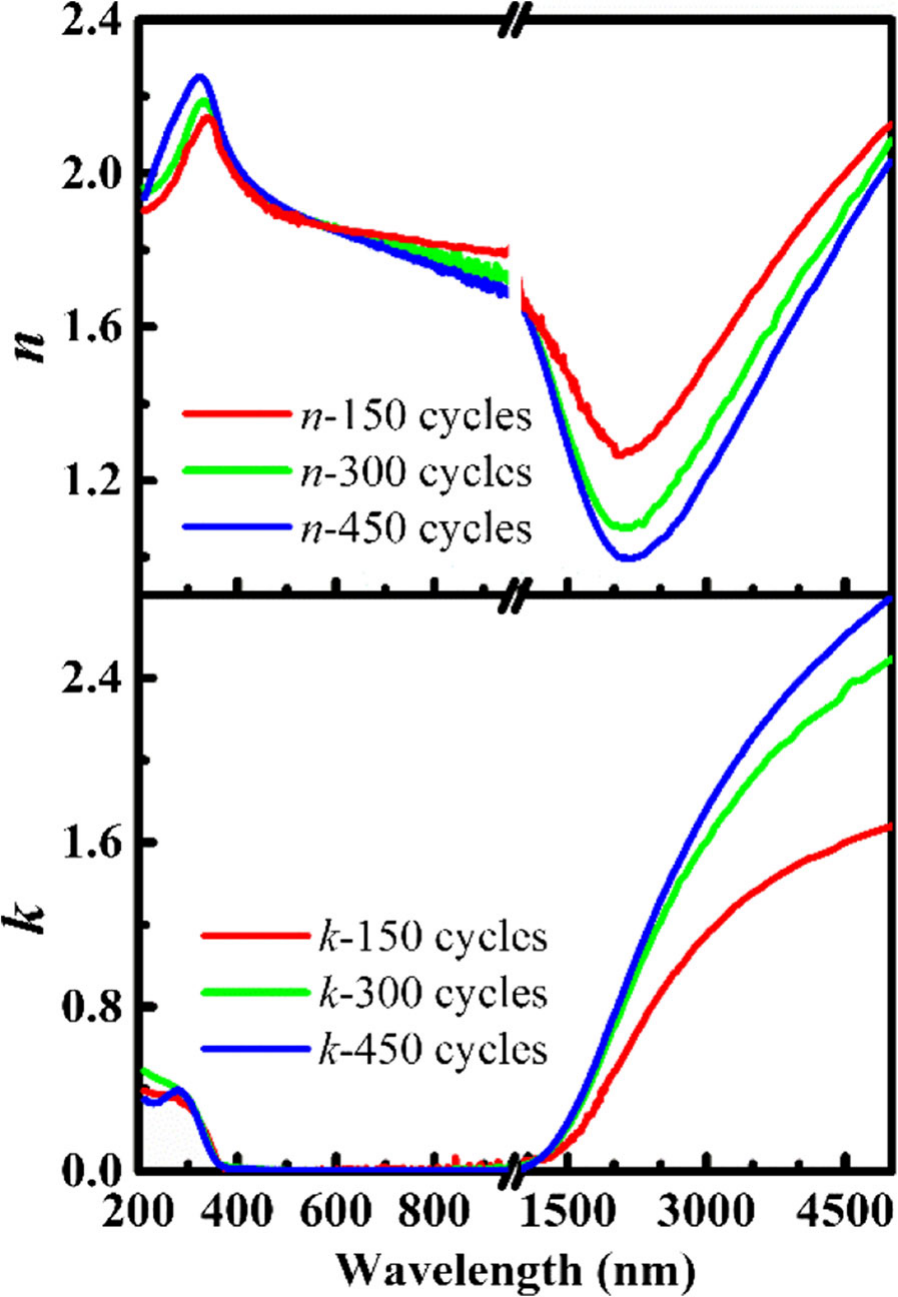
Refractive index (*n*) and extinction coefficient (*k*) were simulated by point-by-point analysis using the data from SE measurements

Here *x* is the number of data points in the spectra, *y* is the number of variable parameters in the model, and “exp” and “cal” represent the experimental and the calculated data, respectively [28]. In the previous report [22], we used the Forouhi-Bloomer (F-B) dispersion model to fit ellipsometry parameters of ZnO at 300–800 nm. Due to the metallic properties of AZO, however, the F-B model is not suitable for AZO films in the whole spectrum from 200 to 5000 nm, which is a model only for single electron transition [29]. Considering the transparency and metallicity of AZO, the Cauchy model is appropriate for the spectrum of 400–800 nm and the Drude-Lorentz model is appropriate for the infrared (1500–5000 nm) [7, 17]. We obtained the thickness and initial parameters of *n* and *k* of AZO thin films from the lowest RMSE of the simulation data as shown in Table 1, where the SEM results were consistent with the SE simulation. Furthermore, a point-by-point analysis [20] was used to calculate the *n* and *k* at the whole wavelength, and the results are presented in Fig. 1. There are two regions of *n* and *k*, which are separated by switching the SE working range. Also, the fitting results can be divided into two regions, i.e., the visible region and the infrared region. In the visible region (210–800 nm), the value of *n* and *k* of AZO was approximate to ZnO for the low percentage of Al. The *n* and *k* in the visible region indicate regular semiconductor properties. The value of *k* is near to zero in the visible range and *n* is thickness-dependent. Here, the thickness dependence was explained by the interface effect [22], which plays an important part in thin films. For the silicon substrate, the interface effect results in the lower permittivity of AZO thinner films in the visible range. However, the trend of *n* and *k* was changed in the infrared region (800–5000 nm). With the increasing of the wavelength, *k* increased from zero, which is the huge difference between AZO and ZnO. The increase of *k* indicated the increase of the film absorption, and the AZO film cannot be used as transparent dielectric material in infrared. There are metallic properties of AZO in the infrared region, which is not only a semiconductor but also a metal material in the infrared region. Moreover, a Hall measurement indicated the bulk carrier concentration of AZO was approximately 1.9 × 10^21^/cm^3^. The high concentration refers to the existence of free electrons, because of Al dopant. An opposite thickness dependence was shown in infrared. The mechanism of the thickness dependence is not the same in the infrared region. The interface effect remains, but the impact is no longer important due to the narrower differences in permittivity between AZO and interface layer while the permittivity of AZO is low in the infrared region. It is assumed that the permittivity of AZO was also influenced by the thickness-dependent crystallinity, which influenced the polarization of AZO thin films.

**Table 1:** Thickness of AZO films simulated by SE and measured by SEM, respectively

Methods	150 cycles	300 cycles	450 cycles
SE (nm)	26.0	50.3	75.6
SEM (nm)	24.8	47.3	75.4

**Fig. 2:**
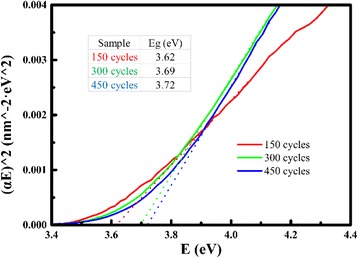
The band gap (Eg) of AZO films by linear extrapolation, where *α* is the absorption coefficient (*α* = 4*πk*/*λ*) and *E* is the photon energy

Furthermore, a linear extrapolation to (*αE*) ^2^ = 0 was used at the absorption edge to obtain the band gap of AZO films in Fig. 2, where *α* is the absorption coefficient (*α* = 4*πk*/*λ*) and *E* is the photon energy [28]. The high energy of the absorption edge of AZO results from the free electron screening effect [16], which suppresses the excitonic absorption. The table in Fig. 2 indicates a blue shift of the band gap (Eg) of AZO from 3.62 to 3.72 eV.

**Fig. 3:**
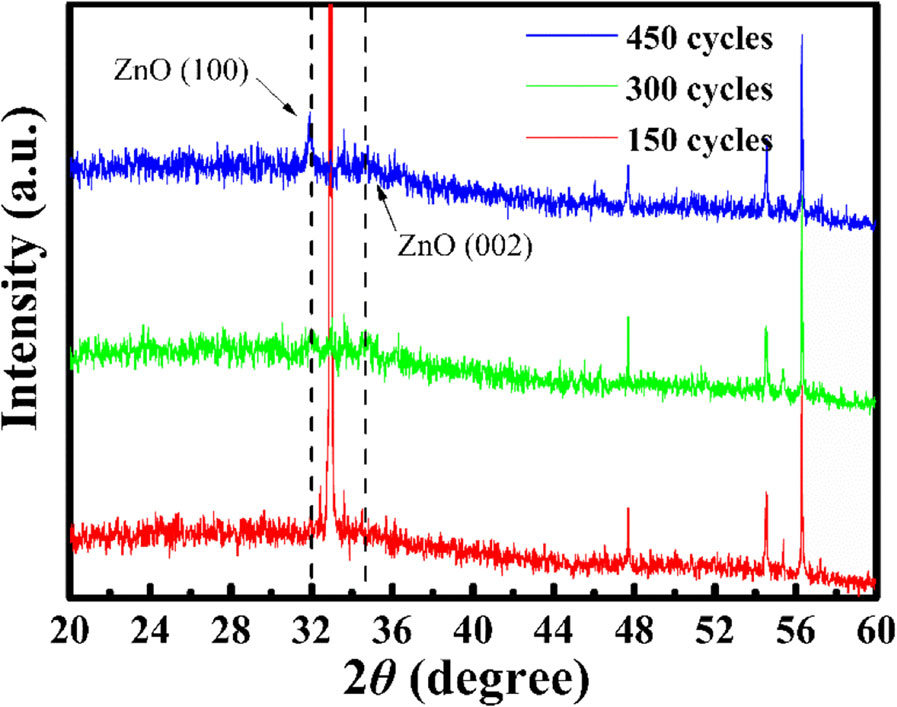
XRD patterns of the AZO thin films with different thicknesses

Moreover, XRD was supposed to measure the crystallinity of AZO films. Figure 3 gives the XRD patterns of the AZO thin films with different thickness. Compared with ZnO films, AZO films are not very crystallographic, as a result of the Al doping. The obvious crystal peak is (100) in the sample with 450 cycles, which represents the hexagonal wurtzite phase of polycrystalline ZnO [30, 31]. Thermal annealing does have an effect on the crystalline property, and this has been discussed elsewhere [7, 9, 10, 22, 32]. The thickness-dependent crystallinity can be used to explain the SE results. The higher crystallinity means the fewer lattice defects, and film stress and strain, which contributes to the blue shift of band gap, higher carrier concentration, and polarization.

In conclusion, the AZO films were not highly crystallized and the crystallinity depended on the thickness, which resulted in a blue shift of band gap and the change in permittivity.

**Fig. 4:**
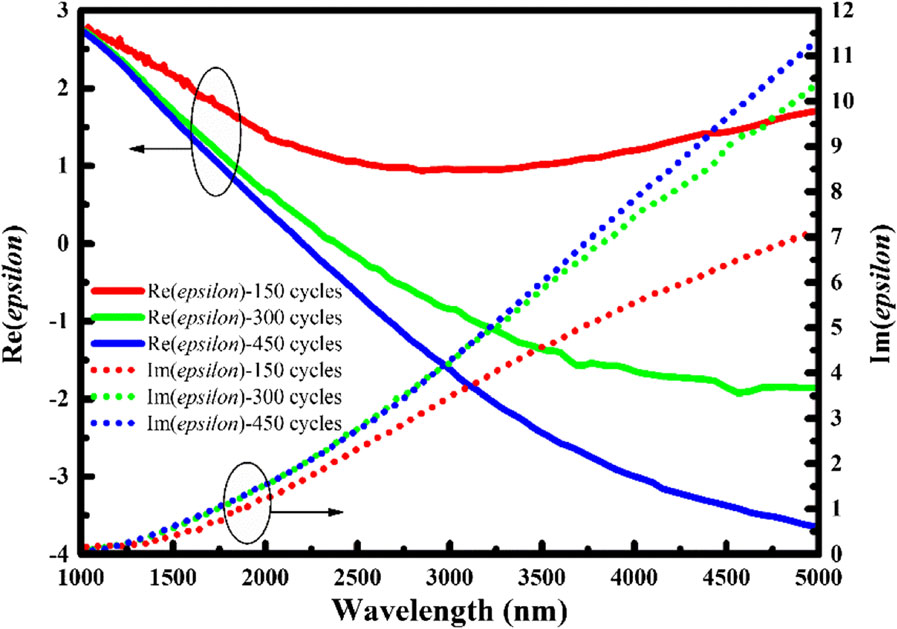
The real part and imaginary part of epsilon of AZO films with different thicknesses, which was calculated from *n* and *k* ($\overset{\sim }{\varepsilon_r}={n}^2-{k}^2+i\ast 2 nk$)

On the other hand, we changed *n* and *k* into permittivity *ε*_*r*_ ($\overset{\sim }{\varepsilon_r}={n}^2-{k}^2+i\ast 2 nk$), and the real imaginary parts of *ε*_*r*_ are illustrated in Fig. 4. The real part of *ε*_*r*_ decreases with the thickness increasing when the imaginary part of *ε*_*r*_ increases. In specific terms, the real part of *ε*_*r*_ is negative in some regions of the spectrum, and the point when the real part of epsilon trends to zero exists. In line with the metallic properties of metal described by the Drude model, the frequency when the real part of epsilon trends to zero is called plasma frequency. Table 2 illustrates that the effective plasma frequency of AZO has a blue shift when the thickness increases. Moreover, for the lower thickness sample, 150-cycle AZO films, the zero point does not exist in the infrared region. In brief, thickness influences the permittivity of AZO, and the real part of epsilon of AZO ultrathin films is always positive. In another word, AZO films could not be regarded as a metamaterial at ultrathin thickness, where the negative real part of epsilon is of importance in plasmonic applications [12].

**Table 2:** Wavelength when the real part of epsilon of AZO thin films is zero in Fig. 4

	150 cycles	300 cycles	450 cycles
Wavelength (nm)	/	2390	2204
Effective plasma frequency (eV)	/	0.519	0.563

**Fig. 5:**
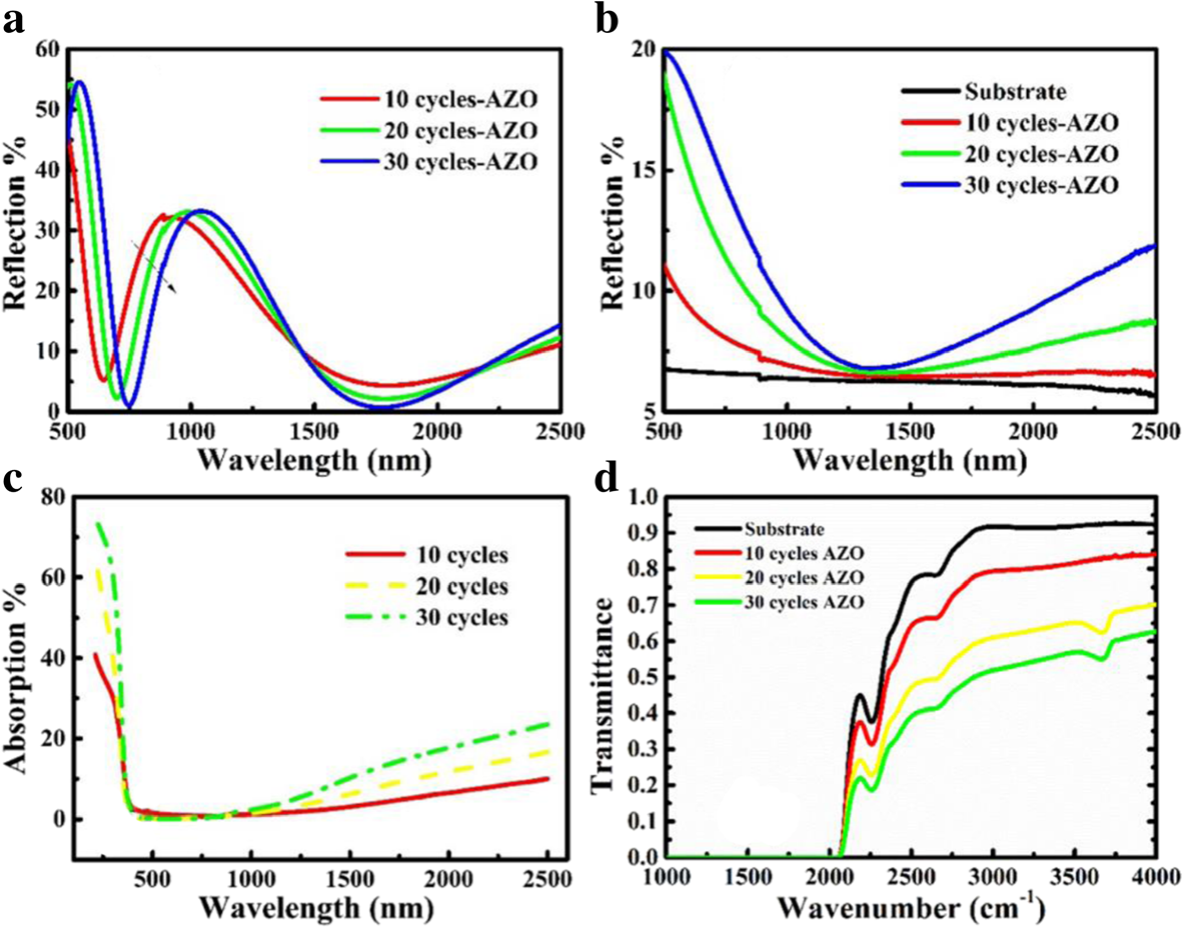
**a** Reflection of AZO films on Si substrate; **b** Reflection, **c** absorption, and **d** transmittance of AZO films on SiO_2_ substrate

Figure 5 illustrates the reflection, absorption, and transmission of investigated AZO films. Figure 5a, b illustrate the reflection of AZO films on Si and SiO_2_ substrates, respectively. It was found that there is higher reflection on the higher thickness of AZO on SiO_2_ substrate. The low reflection of AZO on SiO_2_ substrate in 1000–1500 nm results from low *n* and *k* in Fig. 1. The absorption data in Fig. 5c were calculated from the reflection and transmission. It is assumed that the sum of absorption, reflection, and transmission is equal to 1. The absorption curves in Fig. 5c illustrate that the absorption of AZO films is thickness-dependent in the infrared region, which is consistent to the SE calculation and analysis. The transmission curves in Fig. 5d were measured by FTIR. Between 2500 and 5000 nm (equal to 4000–2000 cm^− 1^), there is lower transmission in the thicker AZO films.

### Near Perfect Absorption Application by Void Cylinder Arrays on AZO Alternative Layers

**Fig. 6:**
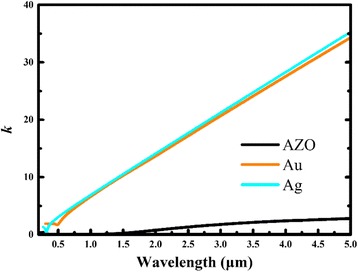
Extinction coefficient k of AZO, Au, and Ag ranges from 0.2 to 5.0 μm [33, 34]

AZO is usually used instead of noble metals as a low-loss plasmonic material in the infrared region [12], but it is also appropriate to build a high absorber in infrared broadband in view of its comparatively lower extinction coefficient, as indicated in Fig. 6.

**Fig. 7:**
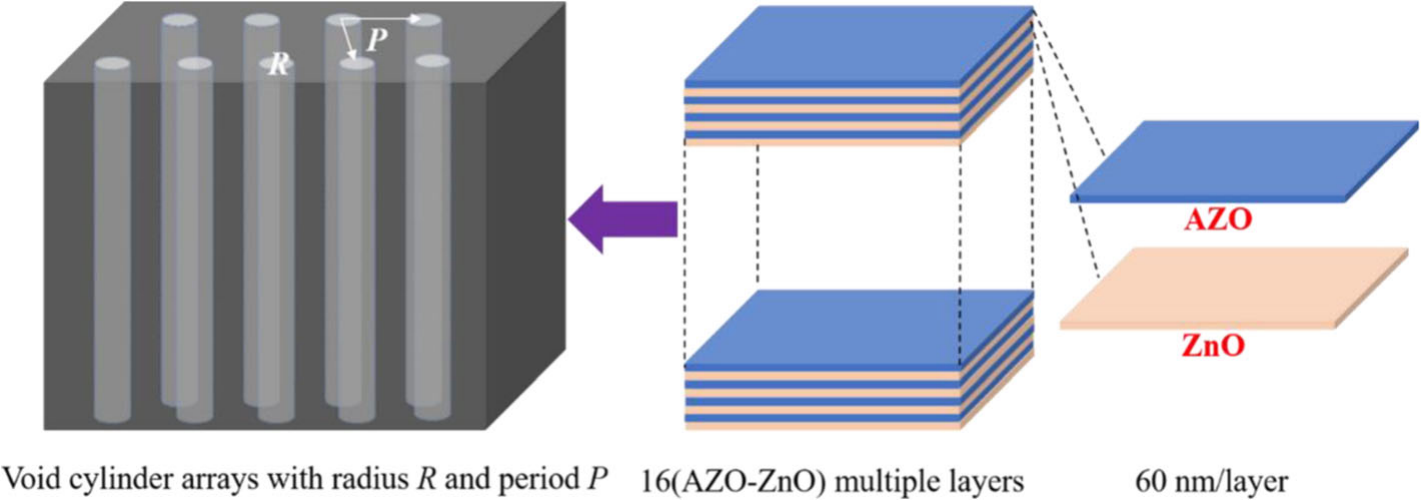
The structure of void cylinder arrays on AZO/ZnO alternative layers. The radius of the void cylinder arrays is *R* μm, and the period is *P* μm. The thickness of 32 layers of AZO/ZnO alternative films is approximately 1.92 μm, each layer being 60 nm thick

In our earlier work [11], 32 layers of AZO/ZnO alternative films were deposited on silicon or quartz substrate by ALD. The thickness of 32-layer alternative films is approximately 1.92 μm, each layer being 60 nm thick. The alternative layers were used to design absorption structures due to near-perfect absorption at ~ 1.9 μm. We took the parameters of AZO thin films from the SE analysis and those of ZnO thin films from our earlier work, then used FDTD solutions as the simulation software to simulate the absorption of the arrays with different parameters. Figure 7 illustrates the absorber structure built by void cylinder arrays on AZO/ZnO alternative layers. The radius of the void cylinder arrays is *R* μm and the period is *P* μm.

**Fig. 8:**
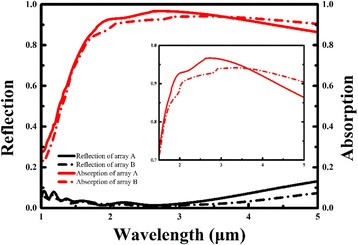
Reflection and absorption of array A and array B

As a result, Fig. 8 presents two kinds of arrays for high absorption and low reflection at a range of between 2 and 5 μm. The specific data are presented in Tables 3 and 4. For array A, the radius is 0.6 μm and the period is 1.8 μm; for array B, the radius is 0.8 μm and the period is 2.0 μm. Array B has broadband absorption of between 2.04 and 5 μm, in which the absorption is more than 0.9. Array A has better absorption than array B in the near-infrared. The negative real part of permittivity of AZO allows the alternating layers to match the phase of all reflected light while the periodic arrays and low permittivity contribute to the infrared broadband.

**Table 3:** Parameters of arrays in Fig. 8

Parameters	Radius (μm)	Period (μm)	The highest absorption
Array A	0.6	1.8	0.967
Array B	0.8	2.0	0.941

**Table 4:** High absorption band of arrays in Fig. 8

Absorption band X (μm)	X_1_ = 0.8	X_2_ = 0.9	X_3_ = 0.9	X_4_ = 0.8	X_4_–X_1_	X_3_–X_2_
Array A	1.61	1.84	4.33	> 5	> 3.39	2.49
Array B	1.64	2.04	> 5	> 5	> 3.36	> 2.96

**Fig. 9:**
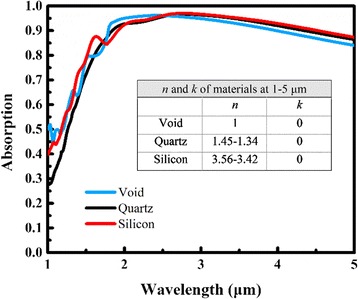
Absorption of array A with different substrates. The insert table shows the *n* and *k* of three substrates, respectively

Figure 9 indicates the absorption of absorber A on different substrates in the infrared region. The void, silicon, and quartz are all transparent in the infrared region. While the refractive index *n* changes from 1 to 3.56, the absorption changes little, which demonstrates the feasibility and applicability of the structure.

## Conclusions

In summary, we examined the thickness-dependent properties of AZO films and designed an AZO infrared broadband absorber. The thickness of AZO films influences permittivity in both the visible and infrared regions. There are two different physical mechanisms, interface effect and thickness-dependent crystallinity, that lead to thickness-dependent permittivity. Furthermore, there is a blue shift for the effective plasma frequency of AZO with an increase in thickness, which does not exist with low thickness (< 25 nm) in the infrared region. These two thickness-dependent properties demonstrate a new method adjusting the thickness to modulate the properties of AZO thin films and indicate that the AZO ultrathin film cannot be used as a metamaterial. Based on AZO permittivity properties, we designed near-perfect infrared arrays by using 32 alternative layers of AZO and ZnO. The negative real part of permittivity of AZO allows the alternative layers to match the phase of all reflected light while the periodic arrays and low permittivity contribute to the infrared broadband. Moreover, the AZO absorber demonstrates feasibility and applicability on different substrates. It is believed that these investigations contribute to a better understanding of the optical properties of AZO thin films in the visible and infrared region for optical and plasmonic applications and that they demonstrate the possibility and feasibility of the AZO absorber at 2–5 μm.

## Abbreviations

ALD: Atomic layer deposition
AZO: Aluminum-doped zinc oxide
F-B: Forouhi-Bloomer
FDTD: Finite difference time domain
FTIR: Fourier transform infrared spectroscopy
RMSE: Root mean square error
SE: Spectroscopic ellipsometry
SiO_2_: Silicon dioxide
XRD: X-ray diffraction
ZnO: Zinc oxide
